# Supplementary material for: Synthesis of C8‐Vinyl Chlorophylls d and f Impairs Far‐Red Light Photoacclimation and Growth Under Far‐Red Light
Source: Physiol Plant. 2025 Nov 19;177(6):e70647. doi: 10.1111/ppl.70647 (PMC12629889; doi:10.1111/ppl.70647)
Supplement: Supplementary file 1 — Table S1: List of strains and plasmids described in this study. Table S2: List of primers used in this study. Figure S1: Multiple sequence alignment of PSII D1 proteins, highlighting residues implicated in tolerance of 8 V Chls. D1 sequences from 8 V‐Chl producing Prochlorococcus spp. (cyan), model WL cyanobacteria that synthesise Chl a only (green), an Acaryochloris strain that primarily makes Chl d (orange), and FR PSII from FaRLiP cyanobacteria (pink), are shown, separated by horizontal lines. Culture collection/accession numbers are used in place of full names, and are ordered as follows: Prochlorococcus marinus MIT 9313, Prochlorococcus marinus MIT 9211, Prochlorococcus marinus subsp. pastoris CCMP 1986 (previously MED4), Prochlorococcus marinus subsp. pastoris CCMP 1986 (previously SS120), Thermosynechococcus vestitus BP‐1 (formerly T. elongautus BP‐1), Synechocystis sp. PCC 6803, Nostoc sp. PCC 7120 (aka Anabaena sp. DCC D0672), Acaryochloris marina MBIC11017, Synechococcus sp. PCC 7335, Altericista leshanensis CCNU0014, Chlorogloeopsis fritschii PCC 9212, Chroococcidiopsis thermalis PCC 7203, Fischerella thermalis PCC 7521 (aka Mastigocladus laminosus Y‐16‐m), Halomicronema hongdechloris C2206. [file PPL-177-e70647-s001.docx]

**Supplementary tables & figures**

| Strain/Plasmid | Properties | Source/Reference |
| --- | --- | --- |
| *E. coli* |  |  |
| NEB^®^ 5-alpha | Cloning strain for pRL277 constructs | New England Biolabs |
| HB101 | Donor strain for biparental mating | Promega |
| *C. thermalis* |  |  |
| WT | PCC 7203 | Pasteur Culture Collection |
| Δ*bciB* | Replacement of central ~700 bp of Chro_1099 with *ermC* from pRL692 in WT, *Em^R^* | This study |
| pseudoWT | Introduction of *ermC* from pRL692 into neutral site in WT genome*, Em^R^* | This study |
| Plasmid |  |  |
| pRL692 | Source of *ermC* cassette, *Sp^R^*, *Em^R^* | Addgene, [1] |
| pRL277 | Cargo vector, *Sp^R^* | Addgene, [2] |
| pRL277[bciB KO] | pRL277 containing regions up- and downstream of Chro_1099 flanking *ermC*, *Sp^R^*, *Em^R^* | This study |
| pRL277[pseudoWT] | pRL277 containing convergent regions of Chro_1103 and Chro_1104 flanking *ermC*, *Sp^R^*, *Em^R^* | This study |
| pRL443 | Conjugal plasmid for mobilization of plasmids to cyanobacteria*, Tc^R^*, *Ap^R^* | Addgene, [3] |
| pRL528 | Helper plasmid for bacterial conjugal DNA transfer, C*m^R^* | Addgene, [4] |

**Supplementary Table 1. List of strains and plasmids described in this study**

[1] Koksharova OA, Wolk CP. 2002. A novel gene that bears a DnaJ motif influences cyanobacterial cell division. *Journal of Bacteriology*. **184**(19):5524-5528.

[2] Cai YP, Wolk CP. 1990. Use of a conditionally lethal gene in *Anabaena* sp. strain PCC 7120 to select for double recombinants and to entrap insertion sequences. *Journal of Bacteriology*. **172**(6):3138-3145.

[3] Elhai J, Vepritskiy A, Muro-Pastor AM, Flores E, Wolk CP. 1997. Reduction of conjugal transfer efficiency by three restriction activities of *Anabaena* sp. strain PCC 7120. *Journal of Bacteriology*. **179**(6):1998-2005.

[4] Elhai J, Wolk CP. 1988. Conjugal transfer of DNA to cyanobacteria. *In* Methods in enzymology (Vol. 167, pp. 747-754). Academic Press.

| Primer name | Sequence (5’-3’) | Restriction site |
| --- | --- | --- |
| bciBKOUpF | GTGCGGCCGGTTAGCTATCTGCGATGATATTGG | EagI |
| bciBKOUpR | CAATTCTTTCAATGACTACACCCCTAGGCACCTCGAGCAATGGTACTAACAATACCCGTC | AvrII, XhoI |
| bciBKODownF | GACGGGTATTGTTAGTACCATTGCTCGAGGTGCCTAGGGGTGTAGTCATTGAAAGAATTG | XhoI, AvrII |
| bciBKODownR | GTCGAGCTCCTTATACAACTACTCTTGATAACC | SacI |
| pseudoWTUpF | GTGCGGCCGGACGGGTATTGTTAGTACCATTG | EagI |
| pseudoWTUpR | CTCTTTTGTTGCTTTGGCAGGCCTAGGCACCTCGAGGAGTGTTAGAAACTAGGCTG | AvrII, XhoI |
| pseudoWTDownF | CAGCCTAGTTTCTAACACTCCTCGAGGTGCCTAGGCCTGCCAAAGCAACAAAAGAG | XhoI, AvrII |
| pseudoWTDownR | GTCGAGCTCCTACAGAAGGAACCCTCATTACTG | SacI |
| EmRF | GTCACTCGAGGCGTGCTATAATTATACTAATTTTATAAGGAGG | XhoI |
| EmRR | CTGCCTAGGTGAGTGAGCTGATACCGCTCGCC | AvrII |
| bciBKOCheckF | GCACCCGGGGTAATTCCGAC |  |
| bciBKOCheckR | CCTAAACTGGAAGCCGCGACC |  |
| pseudoWTCheckF | GTGCATCAATCGATGACAGCGG |  |
| pseudoWTCheckR | CTGAGCCAGCCAAGAACTATGG |  |

**Supplementary Table 2. List of primers used in this study**

Restriction enzyme cleavage sites used for cloning are highlighted in the primer sequence.


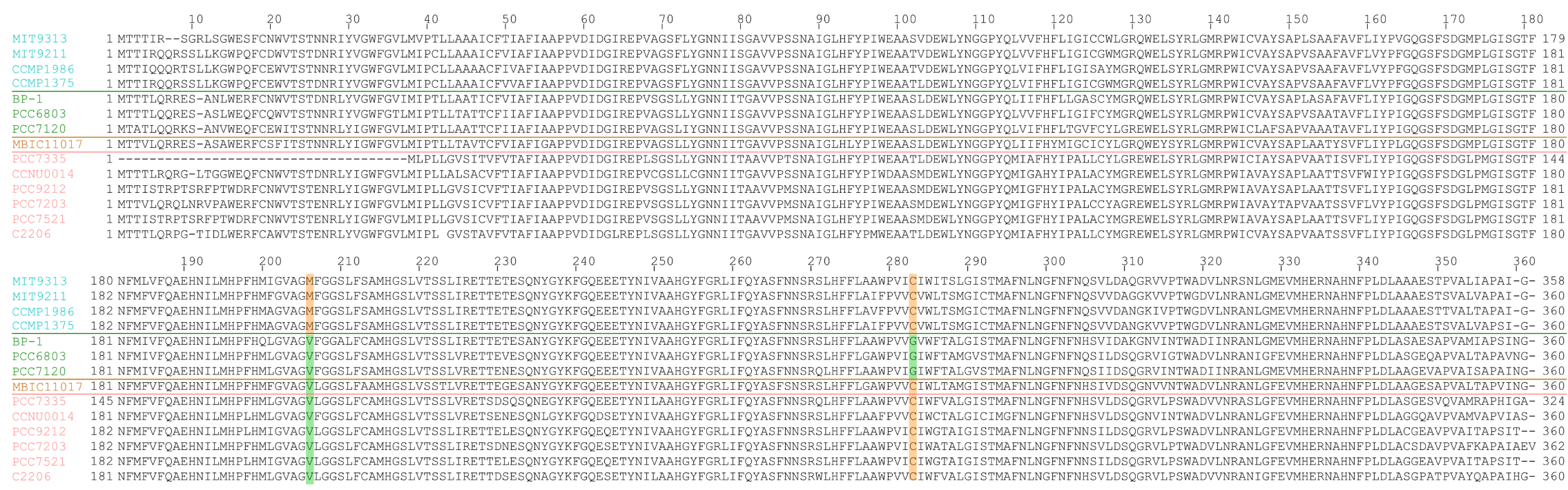


**Figure S1. Multiple sequence alignment of PSII D1 proteins, highlighting residues implicated in tolerance of 8V Chls.** D1 sequences from 8V-Chl producing *Prochlorococcus* spp. (cyan), model WL cyanobacteria that synthesise Chl *a* only (green), an *Acaryochloris* strain that primarily makes Chl *d* (orange), and FR PSII from FaRLiP cyanobacteria (pink), are shown, separated by horizontal lines. Culture collection/accession numbers are used in place of full names, and are ordered as follows: *Prochlorococcus marinus* MIT 9313, *Prochlorococcus marinus* MIT 9211, *Prochlorococcus marinus* subsp. pastoris CCMP 1986 (previously MED4), *Prochlorococcus marinus* subsp. pastoris CCMP 1986 (previously SS120), *Thermosynechococcus vestitus* BP-1 (formerly *T. elongautus* BP-1), *Synechocystis* sp. PCC 6803, *Nostoc* sp. PCC 7120 (aka *Anabaena* sp. DCC D0672), *Acaryochloris marina* MBIC11017, *Synechococcus* sp. PCC 7335, *Altericista leshanensis* CCNU0014, *Chlorogloeopsis fritschii* PCC 9212, *Chroococcidiopsis thermalis* PCC 7203, *Fischerella thermalis* PCC 7521 (aka *Mastigocladus laminosus* Y-16-m), *Halomicronema hongdechloris* C2206.

,
